# The Role of Emotional vs. Cognitive Intelligence in Economic Decision-Making Amongst Older Adults

**DOI:** 10.3389/fnins.2020.00497

**Published:** 2020-05-26

**Authors:** Kanchna Ramchandran, Daniel Tranel, Keagan Duster, Natalie L. Denburg

**Affiliations:** ^1^Department of Internal Medicine, Carver College of Medicine, Iowa City, IA, United States; ^2^Department of Neurology, Carver College of Medicine, Iowa City, IA, United States

**Keywords:** neuroeconomic decision-making, structural imaging biomarkers, emotional intelligence, aging decision competency, fluid intelligence, cognitive reserve

## Abstract

The links between emotions, bio-regulatory processes, and economic decision-making are well-established in the context of age-related changes in fluid, real-time, decision competency. The objective of the research reported here is to assess the relative contributions, interactions, and impacts of affective and cognitive intelligence in economic, value-based decision-making amongst older adults. Additionally, we explored this decision-making competency in the context of the neurobiology of aging by examining the neuroanatomical correlates of intelligence and decision-making in an aging cohort. Thirty-nine, healthy, community dwelling older adults were administered the Iowa Gambling Task (IGT), an ecologically valid laboratory measure of complex, economic decision-making; along with standardized, performance-based measures of cognitive and emotional intelligence (EI). A smaller subset of this group underwent structural brain scans from which thicknesses of the frontal, parietal, temporal, occipital, cingulate cortices and their sub-sections, were computed. Fluid (online processing) aspects of Perceptual Reasoning cognitive intelligence predicted superior choices on the IGT. However, older adults with higher overall emotional intelligence (EI) and higher Experiential EI area/sub-scores learned faster to make better choices on the IGT, even after controlling for cognitive intelligence and its area scores. Thickness of the left rostral anterior cingulate (associated with fluid affective, processing) mediated the relationship between age and Experiential EI. Thickness of the right transverse temporal gyrus moderated the rate of learning on the IGT. In conclusion, our data suggest that fluid processing, which involves “online,” bottom-up, cognitive processing, predicts value-based decision-making amongst older adults, while crystallized intelligence, which relies on “offline” previously acquired knowledge, does not. However, only emotional intelligence, especially its fluid “online” aspects of affective processing predicts the rate of learning in situations of complex choice, especially when there is a paucity of cues/information available to guide decision-making. Age-related effects on these cognitive, affective and decision mechanisms may have neuroanatomical correlates, especially in regions that form a subset of the human mirror-neuron and mentalizing systems. While superior decision-making may be stereotypically associated with “smarter people” (i.e., higher cognitive intelligence), our data indicate that emotional intelligence has a significant role to play in the economic decisions of older adults.

## Introduction

Scientific consensus refers to intelligence as a general mental ability (GMA) that enables complex problem solving, reasoning, experiential learning, the comprehension and generation of original ideas, and the anticipation and adaptation to environmental contingencies (Neisser et al., 1996; Gottfredson, 1997). Amongst these higher order mental abilities, most importantly, intelligence refers to the ability to make sense of the world, and navigate its unknowns, ambiguities and risks constructively. Intelligence is also the ability to anticipate and predict challenges as well as opportunities, and to engage in behaviors that overcome obstacles and create growth (Gottfredson, 2003; Hunt, 2010; Sternberg and Kaufman, 2011; Haier, 2016).

While any construct is only as good as the measures that estimate it, standardized intelligence tests have validated the “g factor” (Spearman, 1904) that unifies various tests of mental abilities pertaining to intelligence, as an emergent property at the top of a pyramid (Carroll, 1993, 2003). “g” has demonstrated wide predictive ability in domains of educational attainment (Brodnick and Ree, 1995), work performance (Schmidt and Hunter, 1998; Gottfredson, 2003) domains of every-day life such as financial decision-making (Agarwal and Mazumder, 2013), and in overall economic and productivity outcomes (Jones, 2011).

With the rapid advancement in neuroscience, we now know that intelligence relies on neural processing of sensory and somatic information (Damasio and Damasio, 1991; Jung and Haier, 2007), thus encompassing and integrating both cognitive and affective domains to address the complexities of life. However, current measures of “g” largely tap putative cognitive domains. Tests that aim to measure more affective, sensory and somatic aspects of intelligence have been developed more recently. Specifically, the construct of emotional intelligence (Salovey and Mayer, 1990), with its accompanying estimates (Baron, 1997; Mayer, 2002), is a relatively recent development. Emotional Intelligence refers to an intelligence construct that involves the ability to read and access emotions to regulate oneself and others, and to utilize emotional information in successfully dealing with life, especially situations that involve social complexities (Salovey and Mayer, 1990; Bar-On, 2006).

Similar to the estimates of the more cognitive aspects of intelligence, estimates of emotional intelligence have found predictive validity in academic (Gil-Olarte Marquez et al., 2006) and workplace (Lopes et al., 2006) achievement, even after controlling for personality and IQ (cognitive intelligence). Emotional intelligence also predicts financial decision-making (Bar-On et al., 2003; Seo and Barrett, 2007), investor behavior (Ameriks et al., 2009) and greater economic self-efficacy (Engelberg and Sjöberg, 2006).

Cognitive intelligence may be classified into two categories: crystallized “gc” and fluid “gf” (Cattell, 1943). “Gf” represents a form of online cognitive processing, associated with inductive reasoning and novel problem-solving (Cattell, 1971) that is relatively independent of prior experience. “Gf” involves the “manipulation of abstractions, rules, generalizations and logical relationships” (Carroll, 1993) in the present moment, while “gc” refers to more offline processing of declarative information accrued by learning, experience and acculturation (Carroll, 1993).

The Mayer Salovey Caruso Emotional intelligence Test (MSCEIT) (Mayer, 2002), an ability and performance measure (as opposed to self-report), posits two higher -order competency factors namely strategic (REA) and experiential (EXP) emotional intelligence that may represent the offline/crystallized and online/fluid aspects of emotional intelligence, respectively. The factor structure of REA is based on the ability to understand emotions based on previous emotional knowledge; to understand emotional complexity based on how emotions may blend or change over time, and the relationships between emotions. It also involves the ability to strategize and manage emotions based on prior emotional experience and knowledge. EXP on the other hand may putatively represent online, fluid emotional processing that involves the ability to accurately perceive emotions in oneself, others and the environment “in the moment.” It also involves the ability to evoke emotions/visceral sensation (vs. emotional recollection) to facilitate and augment thinking (Mayer, 2002; Mayer et al., 2008). Thus, REA and EXP may be taken to putatively represent affective counter-parts to “gc” and “gf”, respectively.

There are age-related declines in “gf” (Horn and Cattell, 1967; Wang and Kaufman, 1993; Ryan et al., 2000) which may leave older adults less adaptive in choosing appropriate strategies (Lemaire et al., 2004), and vulnerable to making inconsistent choices (Tymula et al., 2013) while relying on simpler, less cognitively demanding strategies (Mata et al., 2007; Pachur et al., 2009) in personal financial management (Lachs and Han, 2015). Older adults tend to perform less well than their younger counterparts in cognitively demanding unstructured financial decision environments (Mata et al., 2010, 2012) that tax working memory in the face of multiple concurrent strategic attributes/cues (Fechner et al., 2019). Older adults may also have a positivity bias (Mather and Carstensen, 2003) and overestimate their ability to correctly recall their previous choices (Groß and Pachur, 2019), thus perhaps resulting in cognitive neglect of prior losses in current or future decision-choices.

Additionally, older adults tend to be aversive to ambiguity, especially in the domains of gains rather than losses (Tymula et al., 2013). This discomfort with uncertainty, can lead to heuristic biases, making them less adaptive in decision strategies. There is evidence that “gf” moderates the ability to pick adaptive heuristics in economic decision-making (Michalkiewicz et al., 2018). Hence, discomfort with ambiguity and age-related decline in “gf,” may be some other factors that mitigate adaptive decision-making competence amongst the elderly.

Compromised decision-making (Tymula et al., 2013) thus, is a hallmark of cognitive aging even in healthy older adults making them particularly vulnerable in economic and financial domains. Age-related, brain structural, and functional decline has been implicated in poor trading decisions (Ramchandran et al., 2011). A dispositional effect of hanging onto losing stocks longer than middle aged adults (Feng and Seasholes, 2005) could also affect financial outcomes amongst the elderly. This could be due to the fact that investors, including the elderly tend to be more emotionally affected by loss rather than gain (Kahneman and Tversky, 1979), and their dislike of incurring loss may render them more likely to gamble in the domain of loss (Shefrin and Statman, 1985), hanging onto losing stocks longer than they should (Odean, 1998).

However, skillful individual investors can beat the market (Coval et al., 2005) and both investor sophistication and trading experience can partially attenuate these emotional biases (Feng and Seasholes, 2005). Thus we predict that although older investors may have greater trading experience and financial sophistication than younger investors, they are likely to be biased toward reward-based (Bauer et al., 2013; Eppinger et al., 2013), affective (Worthy et al., 2016) cues in economic decision-making, while neglecting purely rational numerical values. While reward-based cues and numerical values may not be mutually exclusive, an affective bias toward/against loss/gain (Weller et al., 2011) may prevent aging investors from weighing numerical financial values from a cognitively rational/neutral perspective.

Hence, the factors surrounding ambiguity aversion, heuristic, affective, positivity and reward-based biases, coupled with declining “gf” could result in skewed decision choices and compromised economic decision competency amongst older adults. Thus, emotional intelligence, especially its fluid (online processing) aspects, may counter age-related mitigating factors by bolstering cognitive reserve in complex decision-making in older adults, especially in financial and economic domains.

We measured a community dwelling cohort of healthy older adults on a laboratory measure of value-based (reward/punishment) economic decision-making, the Iowa Gambling Task (IGT), known to tap bio-regulatory, neuro-economic processes (Bechara and Damasio, 2005). This task requires (a) cognitively demanding, strategic, monetary selections; (b) convolved with ambiguous, risky cues; (c) that would require tracking information of monetary loss and gain over time; and (d) in a relatively unstructured environment. The version of the task administered in this study involved making choice options that are either low gain/low loss or high gain/high loss. The IGT also requires continuous cognitive flexibility (fluid, online processing) and exploration in sequential decision-making (Ligneul, 2019) over the course of 100 trials, characterizing a “long search” where older adults perform worse than younger adults (Rydzewska et al., 2018).

There is some shared neural architecture (Barbey et al., 2014) between cognitive (Jung and Haier, 2007; Glascher et al., 2009; Gläscher et al., 2010; Haier, 2016) and emotional intelligence (Bar-On et al., 2003; Krueger et al., 2009; Barbey et al., 2014; Operskalski et al., 2015; Smith et al., 2018). A subset of this aging cohort underwent structural imaging allowing us to explore plausible neural underpinnings of the role of cognitive vs. emotional intelligence in economic decision-making decline in healthy aging.

The objective of this research was to (1) examine the relative roles of intellectual vs. emotional intelligence, and (2) their fluid “online” vs. crystallized “offline” components, in economic decision-making in healthy, older adults. We hypothesized that emotional intelligence may bolster cognitive reserve amongst the elderly, and stem decision-making decline. We also hypothesized that fluid aspects of emotional and cognitive intelligence would play a critical role in dynamic, online processing of economic choices. We predicted that fluid processing would significantly impact both (a) the rate of learning in situations of complex choice (as in the IGT) where there is a paucity of information or cues in the initial stages, and (b) in advantageous economic outcomes. Additionally, we also explored plausible neural substrates of the influence of these variables on economic decision-making (IGT).

## Materials and Methods

### Participants

We studied a healthy, community dwelling population of older adults (*N* = 39) with an age range of 55 to 89 years (Mean = 72.69, *SD* = 7.42). Of the 39 participants, 46% were women/54% men. These participants had an education ranging from 11 to 21 years (Mean = 15.60, *SD* = 2.98). For this research, participants were administered the Iowa Gambling Task (Bechara, 2007), the Wechsler Abbreviated Scale of Intelligence (WASI; Wechsler, 1999) and the Mayer Salovey Caruso Emotional Intelligence Test (MSCEIT; Mayer, 2002).

To assess economic, value-based decision-making, we used the *Iowa Gambling Task (IGT)*, which takes about 20 min to administer. This is a laboratory measure of complex decision-making that is sensitive to the integration of affective and cognitive processing and to decision-making deficits (Bechara et al., 2000). Normative data for the IGT exist as well (Bechara, 2007). The IGT measures decision-making under conditions of uncertainty (ambiguity and risk) and has a strong learning component. The task entailed having the participants sit in front of a computer screen, on which were shown four decks of cards labeled A, B, C, and D. The participants could select (click on) a card from any deck. On each choice, the face of the card appeared on top of the deck (the color is either red or black), and a message was displayed on the screen indicating the amount of money the participants had won or lost. At the top of the screen was a green bar that changed according to the amount of money won or lost after each selection. Once the money was added or subtracted, the face of the card disappeared, and the participants could select another card, after a brief delay. The total number of trials was set at 100 card selections. Participants were not told ahead of time how much money they would win or lose as they selected from each deck. Participants were instructed that they were completely free to switch from one deck to another at any time, and as often as they wished. The goal in the game was to win as much money as possible. All money was facsimile, and as participants started sampling the decks, the feedback they received on reward and punishment should have provided cues on identifying good vs. bad decks. Thus, ideally they learned over time that some of those decks were riskier choices (with large rewards yet crippling punishments), but that other decks accumulated financial gain in the long run (smaller rewards with smaller punishments). In this task, the participant always wins some money in every trial, but with some card choices this win is followed sequentially by a loss (within the same trial). Thus, the task encouraged participants to modify their choices and improve decision-making over time. Task uncertainty simulated real life by providing no clues to participants as to when the game might end, and it did so abruptly.

Net raw score (picks from good decks minus bad decks) was the output score on the IGT, and the raw scores were used in the statistical analysis. Additionally, to capture their learning curve from the 1st to the 100th trial, we also computed their raw scores (picks from good minus bad decks) over the progression of the task, in sets of 20 trials. Each set was known as a block and thus, we generated raw scores for 5 blocks from the beginning to the end of the task.

The MSCEIT V.5 (Mayer, 2002) is a standardized ability and performance measure of emotional intelligence addressing various aspects of online and offline affective processing with sub scores for each sub-area of affective processing. The test contains eight different sections of multiple-choice questions that assess various aspects of emotional intelligence. Each section has its own instructions and the participants were encouraged to answer every question, and if unsure to make the best guess. The measure also yielded an overall index score of emotional intelligence (EI), along with two higher-order area sub-scores of Experiential (EXP) and Strategic Emotional Intelligence (REA). Although the MSCEIT yields branch and individual task sub-scores at two levels beneath EXP and REA, we only included the overall EI score and the two area level scores (EXP and REA) for analytic purposes in this research since we were primarily interested in the putative online (fluid-EXP) and offline (crystallized-REA) aspects of emotional intelligence and their respective counterparts for cognitive intelligence. This test was administered to the participants on the Internet and was scored through the vendor's automated system. A consensus scoring system was utilized based on the responses of a normative sample of 5000 individuals across North America. The test has a full-scale internal reliability of 0.91 (Mayer, 2002). It took participants between 30 and 45 min to complete the MSCEIT.

The Wechsler Abbreviated Scale of Intelligence (WASI) (Wechsler, 1999) is a shortened standardized aptitude test of cognitive intelligence (IQ) which provides an estimated composite score of Full-Scale IQ (FSIQ) governed by measures of Verbal Comprehension and Perceptual Reasoning. We administered the version of the WASI containing two subtests namely, Perceptual Reasoning Intelligence (PIQ) and Verbal Comprehension Intelligence (VIQ), taking 30–45 min to accomplish. To measure these domains of cognitive intelligence, the WASI composites scores of Verbal Comprehension and Perceptual Reasoning into a higher-order factor score of FSIQ (Wechsler, 1999). This two-factor model of VIQ and PIQ, had the best goodness-of- fit indices (two factor/ vs. one factor model: Root Mean Square Residual = 0.517/6.104, Tucker Lewis Index = 4.8/124.6), with the normative data (*N* = 2,245), with a correlation of r=0.63 between the two factors (Weschler, 1999). Psychometric research has historically assayed “gc” (based on prior experience and verbal acculturation) with tests of verbal reasoning and “gf” (online problem-solving based on schema, generalizations, and logical relationships) with tests of perceptual reasoning (Cattell, 1943, 1971; Carroll, 1993, 2003; Kaufman et al., 1994). Following this lead, we use VIQ and PIQ as measures of “gc” and “gf”, respectively in this study. The test was administered in-person and scored using algorithms which reflect a normal curve with a mean score of 100. The WASI was standardized using a normative sample of 2,245 individuals, ages 6–90 years, broken into 23 age groups, each of which are scored independently (Maccow, 2011). The WASI has a full-scale internal reliability of 0.94.

Both the MSCEIT and WASI test scores are standardized with Mean = 100, Standard deviation = 15. Each of these tests were administered by trained research assistants in private testing rooms on separate days and thus fatigue in a single sitting was minimized. The MSCEIT data was collected in a separate session from the WASI and IGT, over a two-year period for all 39 subjects. All subjects were comfortable using the computer. Thus, the three main tasks and the MRI data were all conducted in a period that is broadly contemporaneous, within a two-year window. Of the 39 participants in this study, 27 participants (cortical thickness analysis) overlapped with those who underwent structural imaging for a different study. Hence those sMRI data were available for use in our research, and we did not perform new sMRI data collection for the current study (the other 12 subjects were not available for further data collection). All subjects were comfortable using the computer.

Three-dimensional (3D) T1 weighted MRI scans were obtained for 27 of these older participants on a 1.5 Tesla General Electric SIGNA System (GE Medical Systems, Milwaukee, WI), using a spoiled gradient recall sequence with the following parameters: 1.5 mm coronal slices, 40-degree flip angle, 24 ms TR, 5 ms TE, 2 NEX, 26 cm FOV and a 256X192 matrix. Freesurfer (Fischl, 2012) software was used for cortical thickness estimations of all brain lobes and their sub-regions. Since the key neural substrate of the IGT is implicated to be the ventro-medial prefrontal cortex (VMPFC) (Lin et al., 2008; Lezak et al., 2012), cortical thickness estimations of the right, left and bilateral VMPFC were derived using Q-dec, a subcomponent of Freesurfer software. All ROIs were normalized for total cerebral thickness during analysis.

### Group Statistical Analysis

A correlation matrix with multiple comparisons correction was created (Table 1) with all the key variables of economic decision-making (IGT), emotional and cognitive intelligence index and area/sub-scores, age and sex. Table 1 also contains *t*-test values on the top and bottom quartiles of each behavioral variable to examine if they differ significantly. These are reported on the diagonal of the correlation matrix in Table 1.

**Table 1:** Means, standard deviations, and correlations among study variables.

**Variable**	**Mean**	**SD**	**1**	**2**	**3**	**4**	**5**	**6**	**7**	**8**	**9**
1. Age	72.69	7.42									
2. Sex	1.44	0.506	−0.135								
3. Full scale cognitive intelligence	117.62	12.47	0.509^**^	−0.172	4.03^***^						
4. Perceptual reasoning cognitive intelligence	115.13	14.19	0.313	−0.054	0.804^***^	**1.49**					
5.Verbal Comprehension cognitive intelligence	117.38	12.16	0.575^***^	−0.225	0.785^***^	0.382	6.03^***^				
6. Emotional intelligence	92.04	14.58	−0.300	−0.268	0.094	0.015	0.254	**−1.73**			
7. Experiential emotional intelligence	96.14	16.30	−0.465^*^	−0.177	0.039	−0.019	0.126	0.888^***^	−3.19^**^		
8. Strategic emotional intelligence	91.51	12.16	0.019	−0.410^*^	0.109	0.021	0.345	0.815^***^	0.476^**^	**0.721**	
9. Decision-making performance	11.79	40.98	−0.127	−0.203	0.278	0.347	0.150	0.274	0.295	0.179	**−0.43**

* *p < 0.0.5*;

** *p < 0.01*;

*** *p < 0.001 (2-tailed). Multiple Comparisons were accounted for by False Discovery Rate Correction. Uppermost (emboldened) values on the diagonal are t-values of behavioral variables, after patients were divided into top and bottom quartiles based on age. SD, Standard Deviation*.

A set of regressions models (Table 2) were created to examine if economic decision-making was predicted by (Model1) emotional vs. cognitive intelligence index scores; and (Model 2) the fluid (EXP, PIQ) and crystalline (REA, VIQ) area scores of emotional and cognitive intelligence.

**Table 2:** Results of multiple regression analyses predicting decision-making performance in older adults.

**Predictors**	**Adjusted R^**2**^**	**β**
**1. Model 1**	0.092	
Cognitive Intelligence		0.836
Emotional Intelligence		0.704
**2. Model 2**	0.121	
Perceptual reasoning cognitive intelligence		1.017^*^
Experiential emotional intelligence		0.763
Verbal comprehension cognitive intelligence		−0.109
Strategic emotional intelligence		−0.078

N = 39

* *p < 0.05*.

*Dependent Variable: Economic Decision-Making. Control Variable: Sex*.

**Figure 1:**
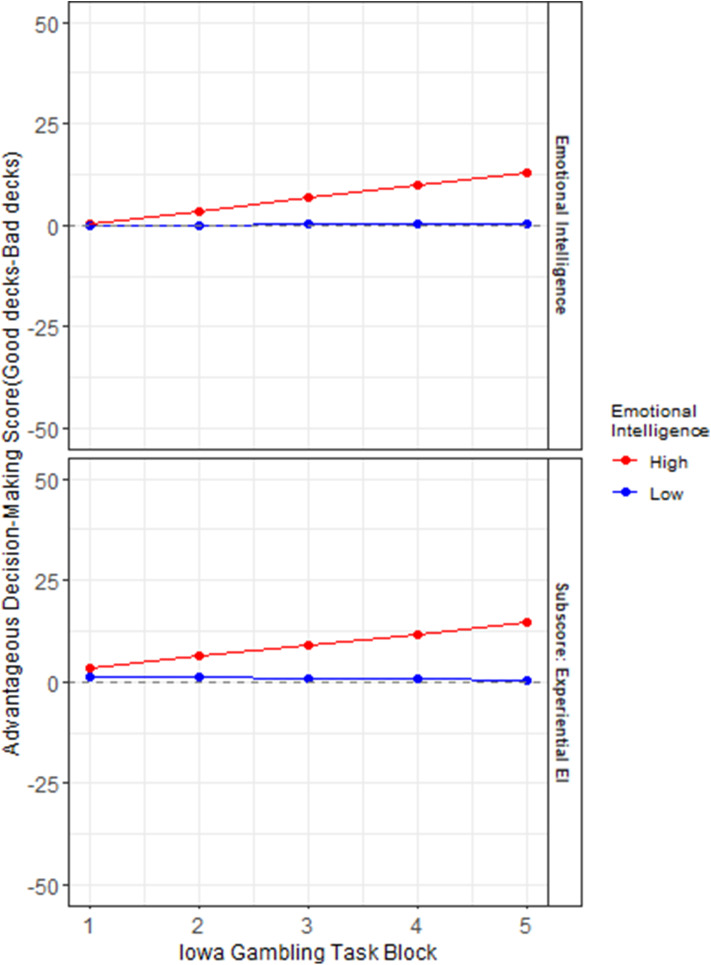
Role of emotional intelligence in rate of learning on the Iowa Gambling Task: The y axes represent advantageous decision-making on the Iowa Gambling Task (IGT), calculated as the number of picks from the bad decks subtracted from the number of picks from the good decks. The x axes represent the progression of the task across trials in 5 blocks (of 20 trials each) from the start to the end of the task. A median split separates high (red) and low (blue) scores on index score of emotional intelligence (EI), and its area sub-score Experiential Emotional Intelligence (EXP).

Another set of hierarchical multi-level regression models (Raudenbush and Bryk, 2002) reported in Table 3, were created to examine if (Model 1) the constructs of emotional vs. cognitive intelligence, and (Model 2) the fluid (EXP, PIQ) and crystalline (REA, VIQ) area scores of emotional and cognitive intelligence, predicted the rate of learning during the course of card selection of 100 trials in the IGT. The outcome variable was the IGT score across 100 trials divided into 5 blocks from start to finish (Figure 1). The emotional and cognitive intelligence index and area scores were median split into (High/low) and used as independent variables in linear mixed effect modeling. A random intercept was assigned to each subject.

**Table 3:** Significant results of hierarchical level models of intelligence and Iowa gambling task block performance.

**Predictors**	**β**	**Standard Error**	**Median value**	**High median demographics**	**Low median demographics**
Model 1: Interaction effects of emotional intelligence^*^IGT block	1.663^*^	0.810	89.741	*n* = 20, *M* = 7, *F* = 13, age 55–85 years	*n* = 19, *M* = 11, *F* = 8, age 66–89 years
Interaction effects of cognitive intelligence^*^IGT block	1.372	0.813	118	*n* = 20, *M* = 10, *F* = 10, age 60–89 years	*n* = 19, *M* = 8, *F* = 11, age 55–80 years
Model 2: Interaction effects of experiential emotional intelligence ^*^IGT block	1.870^*^	0.931	95.020	*n* = 20, *M* = 10, *F* = 10, age 55–85 years	*n* = 19, *M* = 8, *F* = 11, age 66–89 years
Interaction effects of strategic emotional intelligence^*^IGT block	0.464	1.000	90.577	*n* = 20, *M* = 5, *F* = 15, age 55–85 years	*n* = 19, 1 *M* = 13, *F* = 6, age 61–89 years
Interaction effects of verbal comprehension cognitive intelligence ^*^IGT block	0.987	0.969	115	*n* = 20, *M* = 7, *F* = 13, age 60–89 years	*n* = 19, *M* = 11, *F* = 8, age 55–76 years
Interaction Effects of perceptual reasoning cognitive intelligence^*^IGT block	0.174	0.896	117	*n* = 20, *M* = 9, *F* = 11, age 66–89 years	*n* = 19, *M* = 9, *F* = 10, age 55–84 years

*N = 39*,

* *p < 0.05. Dependent Variable: Economic Decision-Making (IGT net score by block). M, male, F, female*.

*Control Variable: Sex*.

Both the above regression analyses (Tables 2, 3) were split into two models each, in order to avoid collinearity between the hierarchically organized index scores and their respective area score variables in models 1 and 2. Combining Models 1 and 2 resulted in variance inflation factors (VIF) that were higher than 10, reflective of high collinearity between variables. Each of the Models 1 and 2, in Tables 2, 3 had acceptable VIFs <6, reflecting an absence of collinearity.

All the 70 ROIs (neural substrates) generated by Freesurfer software were utilized for analyses in conjunction with the intelligence and decision-making variables. This was performed using LASSO (Least Absolute Shrinkage and Selection Operator) regression (Tibshirani, 1996), a penalized regression method that simultaneously performs estimation and variable selection. This is achieved by applying a non-negative penalty term to the magnitude of the beta estimates such that all estimates are shrunk toward zero with some being shrunk all the way toward zero. A larger penalty term performs more shrinkage and thus would indicate a more conservative approach in estimating the association between the independent variables and the dependent variable. An appealing feature of the LASSO is that it can handle high-dimensional settings (i.e., more predictors than observations), such as those arising in this research where Freesurfer has generated 70 cortical thickness regions in a relatively small sample of 27 older adults. Using 3-fold cross-validation to select the penalty term, the LASSO was used as a screening tool to select ROIs amongst the candidate set of 70 regions generated by Freesurfer. Thus, a separate LASSO regression was executed for each of the behavioral outcome variables (EI: REA, EXP; FSIQ: PIQ, VIQ; IGT). For every dependent variable, the most conservative beta estimates of each predictor of the 70 ROIs (that survived the penalization) was selected. These results are presented in Table 4. If more than one ROI survived the penalization for a dependent variable, then the one with the largest beta estimate was chosen as best representing the neural substrate for that construct.

**Table 4:** LASSO regression results.

**Behavioral variables/correlation of brain regions with age**	**RLOFG −0.126**	LRACFG 0.408^*^	**LLFG −0.282**	**RIPFG −0.069**	**LPTFG 0.091**	**RSTFG −0.097**	**LMOFG −0.082**	**RTTFG 0.164**	**LPostCFG 0.106**
EI	β = 1.02								
EI-EXP		β = 6.35	β = 0.06						
EI-REA	β = 1.22			β = 0.66	β = −0.13				
FSIQ						β = −1.27			
PIQ						β = −1.58			
IGT							β = 3.50	β = −162.79	β = −7.69

*The top row contains the brain regions that survived the LASSO regression. Beneath each region is its pure, uncorrected correlation with age*,

* *p < 0.05. RLOFG, Right Lower Occipital Fasciculus Gyrus; LRACFG, Left Rostral Anterior Cingulate Fasciculus Gyrus; LLFG, Left Lingual Fasciculus Gyrus; RIPFG, Right Inferior Parietal Fasciculus Gyrus; LPTFG, Left Posterior Temporal Fasciculus Gyrus; RSTFG, Right Superior Temporal Fasciculus Gyrus; LMOFG, Left Middle Orbitofrontal Fasciculus Gyrus; RTTFG, Right Transverse Temporal Fasciculus Gyrus; LPCFG, Left PostCentral Fasciculus Gyrus*.

A mediation model (Baron and Kenny, 1986) was constructed to examine the causal mediating role of the thickness of the left rostral anterior cingulate fasciculus gyrus in predicting the relationship between experiential emotional intelligence and age.

Another HLM model was used to examine the moderation effects of the thickness of the right transverse tegmental gyrus (RTTG), on rate of learning on the IGT. The thickness of the RTTG was median split (Thicker/Thinner) and used as an independent variable in linear mixed effect modeling. A random intercept was assigned to each subject. Sex was used as a control variable in the regressions reported in Tables 2, 3 and **Figure 5**.

RStudio (RStudio Team, 2015) software was utilized for generation of the Pearson's correlation matrix (Table 1), multiple regression analyses (Table 2), LASSO regression (Table 4) and HLM (Raudenbush and Bryk, 2002) for the moderation analyses. RStudio was also used to execute the mediation model (Baron and Kenny, 1986). Please review the Supplementary Material section containing detailed descriptions of these regressions in Wilkinson-Rogers notation.

## Results

The means and standard deviations of the WASI and MSCEIT scores and sub-scores are reported in the first and second columns of Table 1. This group of older adult participants displays above average intellectual and emotional intelligence scores, as indicated by the normative data of these two instruments (M = 100, SD = 15). The correlations between the index scores of the WASI/MSCEIT and their respective sub-scores retain statistical significance, even after multiple comparisons correction (Table 1), reflecting the robust construct validity and reliability of these two instruments. Of note is that the WASI and MSCEIT index and sub-scores do not cross-correlate with each other or their counterpart fluid and crystalline sub-scores, suggesting that the test construction of these two instruments address disparate aspects of intelligence, perhaps with no overlap.

The core analytic focus of this research was to examine the role of emotional vs. cognitive intelligence in complex economic decision-making processes in older adults, with a secondary exploration of the underlying neuroanatomical substrates.

Table 1 indicates that age is significantly, positively correlated with full scale cognitive intelligence. Age is also significantly positively correlated with its verbal comprehension sub-score (VIQ), which is putatively represented by crystallized intelligence (Kaufman et al., 1994). While fluid intelligence (PIQ) is hypothesized in the literature to decline with age, the two are not significantly correlated in our dataset (Table 1).

However, the fluid counterpart of emotional intelligence (EXP), significantly declines with age. The IGT does not significantly correlate (after multiple comparisons correction) with any of the measures in Table 1.

We were interested in separating the role of EI vs. IQ in economic decision-making and performed additional analyses to that effect. To explore how each of these two modes of intelligence predict performance on a laboratory measure of economic decision-making (IGT), while controlling for the other, we executed two separate multiple regressions (Models 1,2: Table 2) with IGT net score as the outcome measures. The predictors in Model 1 were FSIQ and EI index scores. In Model 2, the fluid intelligence sub-scores of PIQ, EXP, and the crystallized intelligence sub-scores of VIQ and REA were the predictor variables. Sex was a control variable in both Models 1 and 2. Please see Supplementary Material section of Wilkinson-Rogers notation for model details.

The results (Table 2), indicate that PIQ (β = 1.017, *p* < 0.05), the measure of “gf” significantly predicts IGT performance.

The IGT's ecological validity lies largely in its incorporation of ambiguity and risk/reward in financial choice options which the decision-maker must learn to navigate as the task progresses. One goal was to explore how the cognitive and affective aspects of intelligence might influence the rate of learning in discerning patterns of risk from prior wins and losses in making current advantageous monetary choices. To examine the rate of learning, monetary selections in the IGT (number of picks from good decks minus bad decks) across the 100 trials were sorted into 5 blocks (20 trials in each block), representing the progression of the task from start to finish (Blocks 1–5).

Table 3 indicates the significant results of the two hierarchical level models (HLMs) with IGT performance (by deck) as the outcome measure. The median splits (High/low) of (Model 1) EI and FSIQ, (Model 2) EXP, REA, PIQ, and VIQ are predictor variables. Each model controlled for its respective counterparts in cognitive intelligence and sex. The interactions of advantageous decision-making by block X emotional aspects of intelligence significantly predict rate of learning on the IGT in each of the two HLMS, the highest being that for EXP (β = *1.87, p*< *0.05)*. A visual display of these HLMs in Figure 1 indicates that in a median split, those low in the fluid aspect of emotional intelligence (EXP) display the most impaired rate of learning across decks, from start to finish on the IGT. This is followed by low overall index score of EI.

It is in the first few blocks that the greatest ambiguity is presented to participants, when, through trial and error, they are required to learn to distinguish between the good and bad (risky) decks.

It is in these early blocks of the IGT that higher emotional intelligence scores predict a superior rate of learning, whose steady small gains are gradually accrued in putatively greater financial capital by the end of the task, as is visually apparent in Figure 1. This analysis suggests that the affective (rather than cognitive) aspects of intelligence, and especially its fluid aspect, significantly predict the rate of learning on this financial decision-making task under conditions of risk and ambiguity.

In a quest to map some of the neuro-anatomical underpinnings of these behavioral results, the structural imaging correlates of these behavioral variables on a smaller subset (*N* = 27) of this sample were collated using a LASSO regression (see Methods section for details). The results of the LASSO regression presented in Table 4, displays the regression weights of the significant cortical thicknesses of ROIs that that survived the penalty system of the LASSO method, and that were associated with each behavioral variable. Thus, the overall EI index score, was predicted by the cortical thickness of only one ROI, namely, the right lower occipital fasciculus gyrus (RLOFG). On the other hand, three different ROIs survived the penalty system of the LASSO regression for the IGT. In this case, the ROI with the highest regression weight, namely the cortical thickness of the right transverse temporal fasciculus gyrus (RTTFG), is taken to be predictive of IGT performance.

**Figure 2:**
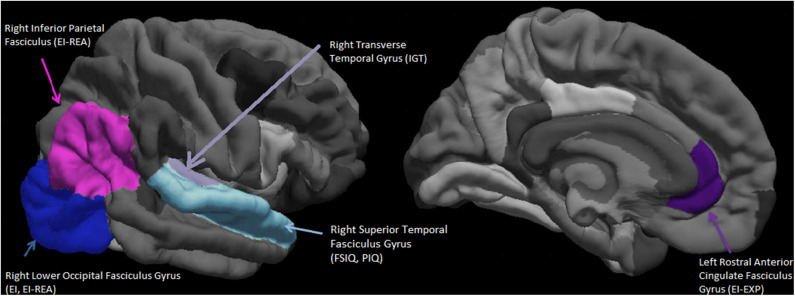
Visual representation (on the Freesurfer software template) of the average cortical thicknesses of the significant Regions of Interest (ROIs). These are predictive of emotional intelligence index score (EI), its area sub-scores Experiential Emotional Intelligence (EXP), Strategic Emotional Intelligence (REA); Full scale cognitive intelligence index score (FSIQ), its sub-score Performance Intelligence Quotient (PIQ), and the IOWA Gambling Task score. *N* = 27.

Seen in Figure 2 is a visual representation of the average cortical thicknesses of the significant ROIs of this population on the Freesurfer software template. The index score for cognitive intelligence and its fluid sub-score of PIQ are both predicted by the thickness of the same ROI, namely the right superior temporal fasciculus gyrus (RSTFG-Table 4, Figure 2). Similarly, both the index score for emotional intelligence (EI) and its crystallized emotional intelligence sub-score of REA are best predicted by the thickness of the RLOFG (Table 4, Figure 2).

Note that no ROI survived the penalty system of the LASSO regression for VIQ (crystallized intelligence counterpart of cognitive intelligence).

Also included in Figure 2 is the right inferior parietal fasciculus gyrus (RIPFG), which has the second highest regression weight associated with REA (Table 4).

The ROIs of the right superior temporal gyrus (FSIQ, PIQ) and the right inferior parietal fasciculus gyrus -Brodmann's areas (BA) 39, 19 (REA) and right lower occipital fasciculus gyrus-BA 17, 18, 19 (EI, REA), confluence around a section of the brain (parietal temporal junction-TPJ) associated with theory-of mind (Schurz et al., 2014); as is the medial prefrontal cortex -BA 24, 32 (LRACFG). These set of regions (BA 24, 32, 39, 37, 18, 19, and 17) are also part of the P-FIT network associated with cognitive intelligence (Jung and Haier, 2007). They represent visuo-parietal association cortices (BA 39, 37, 18, 19) involved with the organization of multi-modal perceptual/sensory information and the frontal limbic areas (BA 24, 32) (Schurz et al., 2014).

**Figure 3:**
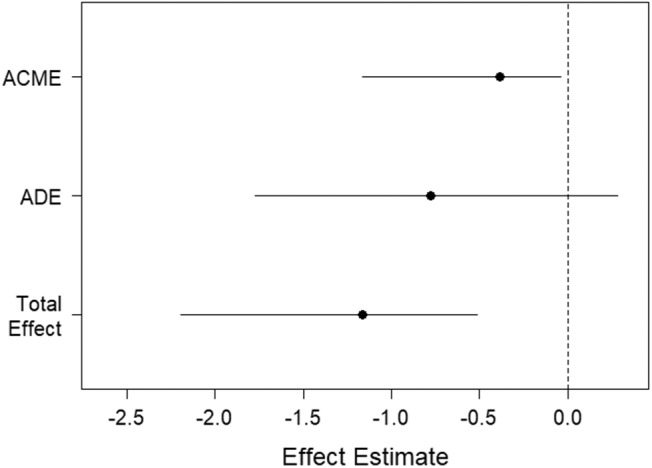
Visual representation of causal mediation of the Rostral anterior cingulate fasciculus gyrus (LRACFG). The Y axis represents the Average causal mediated effect (ACME); the Average direct effect (ADE- residual effect after mediation) of age on experiential emotional intelligence (EXP); and the total effect of the ACME and ADE. The X-axis represents the regression weights (effect estimates).

**Figure 4:**
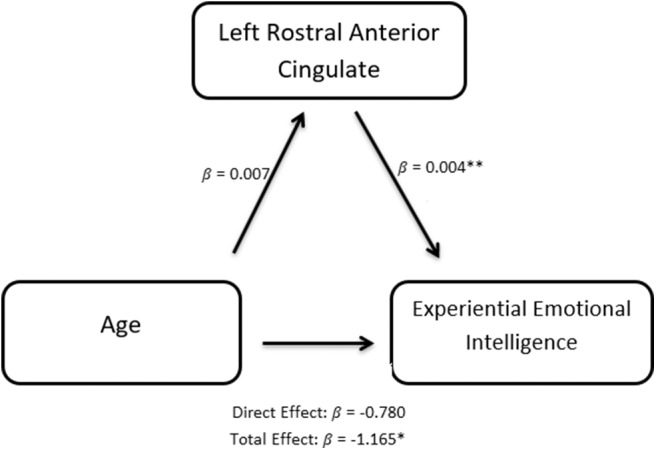
Path diagram of the causal mediation of the Rostral anterior cingulate fasciculus gyrus (LRACFG). Given the significant negative correlation between age and EXP (Table 1), and the negative direction of the regression estimates (Figures 3, 4) between LRACFG and EXP, it appears that those amongst the older aging adults with thinner LRACFG, tend to have better preserved EXP.

Since EXP best predicts the rate of learning on the IGT (Table 3, Figure 1), we explored aging effects of EXP on its neural substrate Left Rostral Anterior Cingulate Gyrus (LRACFG) (Table 4, Figure 2) in a mediation analysis. The thickness of the LRACFG, partially mediates (33%) the relationship between age and experiential EI (EXP) (Figures 3, 4).

The right transverse temporal fasciculus gyrus (RTTFG) emerges as a partial neural substrate for decision-making performance (Table 4). While the thickness of both the left medial orbital frontal gyrus (LMOFG) and the left postcentral fasciculus gyrus (LPostCFG), survive the penalty system of the LASSO regression in predicting IGT score, the regression weight of the TTFG is several-fold higher than the other two regions (Table 4).

**Figure 5:**
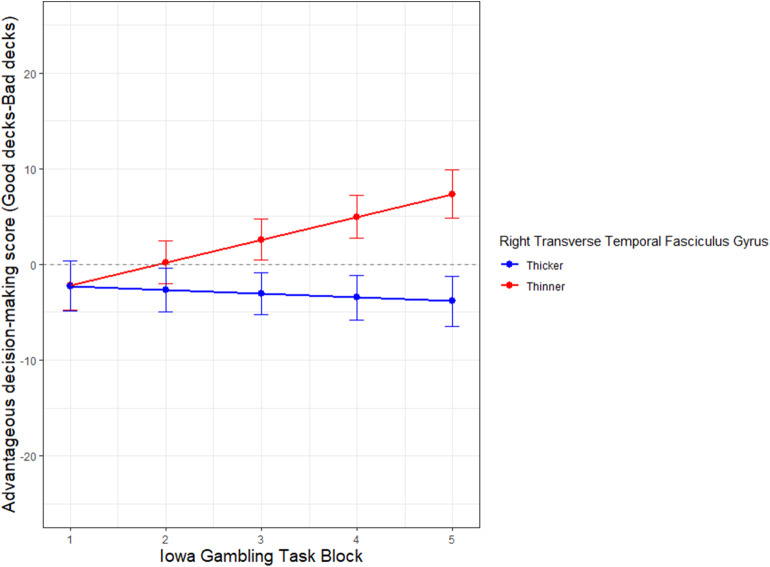
Neural substrate that moderates decision-making performance in aging adults. The X axis represents Iowa Gambling Task (IGT) task performance in 5 blocks (1–5) of 20 trials each, from start to finish. Y axis represents IGT score. The cortical thickness of the neural substrate, right transverse tegmental gyrus (RTTFG), is median split into thick (blue) and thin (red). Control Variable: Sex.

The thickness of the RTTFG also moderates the rate of learning on the IGT as the trials progress from start to finish (Figure 5). The interaction of thickness of RTTFG ^*^IGT block (β = −2.778, SE = 0.9906, *p* < 0.001), after controlling for sex, significantly predicts IGT performance. The median ratio of the RTTFG to total cerebral thickness is 0.191. The demographic distribution of those with thinner cortices is *N* = 14, Age range 61–81 years, 10males, 3 females; while those with thicker cortices is *N* = 14, age range = 60–89, 5 males and 9 females. While all participants start the IGT with loss-making choices, those with thinner cortices (median split) of the RTTFG learn to make advantageous choices over time, while those with thicker cortices of the RTTFG do not. Note that the oldest subjects tend to have thicker cortices (median split). The results are not significantly altered with or without controlling for sex.

## Discussion

To summarize, while the construct of intelligence is considered to be stable over a lifespan, our results indicate that even within a cohort of independent, healthy, community-dwelling older adults, (1) there may be significant individual differences in cognitive and emotional aspects of intelligence that impact their economic decision-making competence; (2) this may be modulated by declines in fluid rather than crystallized aspects of cognitive and emotional intelligence; and (3) these individual differences may be mediated and moderated by structural changes in the brain across the lifespan. We discuss the implications of these results in the context of the neurobiology of aging and the anatomical substrates of cognitive and emotional intelligence.

Theory of mind, referring to insight into the mental states of oneself or others, could putatively be associated with emotional and social intelligence. Cortical thickness data in a subset of this group, reveal an overlapping patchwork of brain regions associated with theory of mind (involving right parietal and right occipital cortex), and the left anterior cingulate (Schurz et al., 2014) as a potential neural substrate for emotional intelligence.

The right superior temporal cortex emerges as a potential neural substrate for cognitive intelligence and its fluid facet (perceptual IQ), while the right transverse temporal gyrus emerges as a potential neural substrate for this economic decision-making task. In this group with structural imaging data, a thinner cortex appears to be predictive of superior performance. The thickness of the left rostral anterior cingulate cortex mediates the relationship between age and experiential emotional intelligence, such that older adults with thinner cortices of LRACFG tend to have higher experiential intelligence. In the case of decision-making performance, those aging adults with thinner right transverse temporal gyri tend to learn with experience in making better economic choices over the course of the task, while those with thicker cortices do not.

Research on intelligence includes proponents of multiple intelligences, while others have proposed that the field is best served by a unitary construct of intelligence requiring the development of superior measures that assay its various components. While Spearman's concept of “positive manifold” (Spearman, 1904) proposed that all estimates of mental abilities positively correlate with each other, this is true in our dataset only for the relationships within the index scores and their respective sub-scores of IQ or EI; but not between the scores/sub-scores of EI and IQ, given that questions on the MSCEIT almost exclusively tap emotional and social domains, which the WASI does not. Hence the absence of “positive manifold” in our research, cannot be interpreted as measuring two different kinds of intelligences.

The literature has been mixed in the relative contribution of EI and IQ to performance on the IGT. Recent studies utilizing the MSCEIT has suggested that individuals low in EI tend to misappraise physiological signals in risk-taking (Yip et al., 2019), and perform poorly on the IGT, a “hot” task with physiological arousal of somatic markers in comparison to a decision-making task that was relatively cognitively “cool” and that did not tap affect (Checa and Fernández-Berrocal, 2019). Cognitive intelligence in these studies, did not significantly predict outcome on the IGT. Training on EI has also indicated gains in performance on the IGT (Alkozei et al., 2019), while cognitive intelligence is considered to be a less malleable construct.

IQ and not EI predicted IGT performance in research utilizing a self-report measure of EI and a measure of verbal IQ in young adults (<25years) (Demaree et al., 2010). More similar to our research, a study utilizing the WASI and three different measures of EI (including the MSCEIT) that were analyzed simultaneously while controlling for IQ, found that cognitive and not emotional intelligence (controlled for EI) predicted IGT performance in a younger cohort (age:18–45) (Webb et al., 2014). In our study in older adults, using some of the same measures, it is the fluid aspects of cognitive intelligence namely perceptual reasoning that predicts IGT performance.

It is possible that relatively preserved emotional intelligence augments aging decline of “gf,” and thus EI and especially its fluid component (EI-EXP) may prove to be more critical in learning across the stages of financial decision-making (after controlling for IQ) within an aging context, as our study suggests. This is supported by a neural substrate, our finding that the thickness of the LRACG mediates the relationship between age and EI. The anterior cingulate, a paralimbic region associated with the midbrain dopamine neuron system's risk/reward processing, is also linked to compromised financial decision-making in older adults due to aging decline in executive control, reward processing and default mode functional connectivity networks (McCormick et al., 2019). Aging structural/functional decline of this region could impact EI's influence on decision-making on the IGT by biasing older adults toward immediate monetary reward (McClure et al., 2004). This region is subsumed within the VMPFC, classically implicated in the affective aspects of IGT performance (Bechara et al., 2000), and implicated in decision-making decline in older adults (Halfmann et al., 2014) on the IGT. The VMPFC, that classically integrates both affective and cognitive inputs, is shown to be insensitive to risky choices in the domains of both gain and loss (Weller et al., 2007), and hence, aging decline of the anterior cingulate could compromise the contributions of both emotional and cognitive intelligence to IGT performance.

The distribution of brain regions (cortical thickness) in relation to the intelligence variables in Figure 2 builds an interesting picture in the light of prior findings. The RSTFG (containing a section of the arcuate fascicule) has been previously implicated with perceptual organization and working memory index of WAIS-III (Glascher et al., 2009), supporting our results of both PIQ and FSIQ mapping on to the same region in this aging population. No region survived the robust penalty system of the LASSO regression in relation to VIQ, though the STFG associated with FSIQ (Basten et al., 2015) would subsume the Wernicke's area, associated with tonal/speech processing and comprehension.

Prior lesion research has implicated the PFC, especially the VMPFC in self-reported EI (Bar-On et al., 2003); and it's sub-regions, the DLPFC and the VMPFC with EI-EXP and EI-REA of the MSCEIT, respectively (Krueger et al., 2009). However, in our healthy aging cohort, those brain regions associated with EI -Figure 2 (and its sub-scores of REA and EXP) map on to the posterior-anterior progression (Sitartchouk and Evans, 2017) of intellectual neural processing postulated by the P-FIT (Jung and Haier, 2007), although this distributed network of brain regions was developed in the context of “g” and cognitive intelligence. Thus, both EI and its REA sub-score are associated with posterior regions of the right hemisphere in this dataset, representing the association/multi-modal sensory cortices, the fusiform and extra-striate cortex (imagery, visual recognition and elaboration) and BA22 (extending syntax of auditory information). All of these represent Stage 1 of processing of intellectual information proposed by P-FIT (Colom et al., 2010). Stage 2 processing proceeds to the inferior parietal lobule (EI, REA) and the planum temporale (LPTFG-Table 4) containing a section of the arcuate fascicule, involving the abstraction and integration of multi-modal sensory information from Stage 1, including speech, auditory and visual comprehension. This section of the superior temporal gyrus around the planum temporale also forms a key “imitation” node of the frontal-parietal human mirror neuron system providing both visual input and serving as a hub of interpreting “intention of action” in theory of mind (Iacoboni and Dapretto, 2006; Schurz et al., 2014). Thus Stage 1 and 2 may represent the process of crystallization of affective/sensory information as per the latent construct of strategic emotional intelligence and the regions associated with these stages have also been previously associated with emotional intelligence (Barbey et al., 2014). Thus, the white matter tract of the arcuate fascicule (contained in both STFG, angular gyrus, planum temporale) may play a significant role in connecting the posterior and anterior (frontal) sections of the brain in the 3rd stage of intellectual processing for evaluation and problem-solving. The fluid component of EI-EXP is associated in our dataset with the left anterior cingulate (Figure 2, Table 4—LRACFG), representing a more fluid component of emotional intelligence (i.e.) emotional regulation-attentional flexibility, error detection and inhibition of pre-potent response. The anterior cingulate (BA 32) also represents Stage 4 of intellectual processing as per the P-FIT for generating alternatives and response selection from Stage 3 solutions (Jung and Haier, 2007; Colom et al., 2010). Thus, these stages of intellectual processing that are postulated to involve bi-directional, hierarchical, parallel processing (Haier, 2016), are applicable to the domain of emotional intelligence as well, in our population of older adults. A recently developed neuro-cognitive model for emotional intelligence postulates a distributed network of regions (Smith et al., 2018) with shared architecture attributed to the P-FIT and the somatic marker hypothesis (Bechara and Damasio, 2005). This points to a unitary construct for intelligence combining sensory, somatic, affective and cognitive components. Hence, it also calls for the development of intelligence estimates that combines all these elements into a single set of measures.

The orbito-frontal (OFC)/ventro-medial prefrontal regions have been classically implicated in IGT (Lezak et al., 2012) performance and especially in the aging context (Denburg et al., 2005; Halfmann et al., 2014). Although the OFC/VMPFC along with the postcentral fasciculus gyrus (association sensory cortex) are implicated as somatic markers of neuroeconomic behavior (Bechara and Damasio, 2005; Damasio, 2009), in our aging cohort, the RTTFG's prediction of IGT performance is significantly higher than the former two regions (Table 4). The primary auditory cortex (RTTFG) associated with the IGT in this dataset and being subsumed within the RSTFG is significant in that auditory perception is a second -order factor of “g” as proposed by J. B. Carroll's three stratum theory of intelligence (Carroll, 1993, 2003). The TTFG folding inward toward the medial temporal lobe and insula has strong connections with these regions including the amygdala, hippocampus and Para hippocampal gyrus. Hence this region receives substantial somatic, sensory, affective and memory-related information, and not just in the auditory context and has been linked to emotional intelligence (Smith et al., 2018) as well, specifically in the context of affective prosody (Belyk and Brown, 2013).

The RTTFG can discriminate between rapid, fine-grained frequency changes of spectral, timbre and temporal sound processing (Samson and Zatorre, 1994; Zatorre and Belin, 2001). This fine-tuned, nimble flexibility may extend to non-auditory, multi-modal, higher-order processing as well, given the wealth and variety of information this gyrus has access to. Thus, the RTTFG may possess the dynamic capabilities to process the IGT which requires quick, fine-grained probabilistic discriminatory choices, sequential (trial by trial) search memory of loss versus gain information and continuous learning over extended (100) trials. The IGT provides auditory feedback (simulating a gambling casino) with different sounds for financial loss and gain, which in older adults may preferentially activate the RTTFG's discriminatory processing. This may be a more parsimonious explanation of this region's implication in IGT performance.

Given that the thickness of the RTTFG moderates quality of decision-making on the IGT across trials; and that a thinner RTTFG predicts better performance, it may be that large inputs of somatic/sensory/affective information may overwhelm financial decision-making in the older adult. Perhaps a thinner auditory cortex's role in superior performance in the IGT may be a function of early developmental pruning, thus optimizing information flow from somatic cues with decision quality. The central acoustic system in the brain is subject to neuroconstructivist (Westermann et al., 2007) cellular and synaptic plasticity associated with sensory exposure and experience (Sanes and Bao, 2009; Yin et al., 2018). Functional activation in the developmental auditory cortex has also been linked to higher language related cognitive scores (Deshpande et al., 2016). In the developmental context, cortical functional activation in acoustic tasks is negatively correlated with cortical thickness, suggesting that cortical thickness and function are likely linked to early pruning of exuberant connectivity (Anurova et al., 2014). All of these suggest that developmental pruning of the RTTFG may be linked with higher rate of learning on the IGT. It is also of note that in our sample, those with thinner RTTFG cortex (higher rate of learning on the IGT) tend to be younger (60–80 years of age), while those with thicker RTTFG cortex (lower rate of learning on the IGT), tend to be older (61–89 years of age) by almost ten years. This may be an artifact of our small sample and we interpret these findings prudently. While our results may be counter-intuitive to the finding that cognitive impairment is linked with cortical thinning (Pacheco et al., 2015), our research is based on a cross-sectional sample where subjects are not followed longitudinally. Importantly, this result pertains to the rate of learning on the IGT, and not to IGT outcome score itself. Hence, our result cannot be treated as clinical evidence of either cognitive impairment or reserve.

One of the major limitations of this research is that our evidence is purely correlational and given a relatively small sample size, these exploratory results provide cues to further investigation of the factors that drive economic decision-making amongst the elderly. Another limitation is that we did not have a younger comparison group with similar measures, that would have allowed us to more thoroughly investigate the role of fluid and crystalline affective vs. cognitive intelligence on economic decision-making.

Although our neuro-anatomical results are interpreted prudently given the small sample, we are intrigued by the potential role of the TTFG and the superior temporal lobe in neuro-economic behavior, as having emerged in this study, as potential regions of interest to be researched in the future. While the IGT has been featured in over 800 studies as an ecologically valid measure of complex economic decision-making (Lezak et al., 2012), its predictive validity is restricted in this study due to the lack of real-world financial measures (Lichtenberg et al., 2018) of these participants that we could have cross-compared our data with.

One strength of these data, although possessing a modest sample size, lies in its inclusion of community dwelling, independent, active, healthy aging adults as old as 89 years. An additional strength has been the combination of both psychological and neuroimaging data that have yielded promising results. The use of the LASSO regression method facilitated the simultaneous exploration and robust examination of large numbers of brain regions relative to a small sample, in association with each behavioral variable.

Our results substantiate the role of fluid (“online processing”) aspects of cognitive and emotional intelligence in bio-regulatory processes that influence economic decision-making through rapid somatic cues that signal impending loss or gain in real-time (Bechara and Damasio, 2005). Additionally, the mediating and moderating effects of neural substrates on fluid aspects of emotional intelligence, age and decision-making in our study, have implications for (1) affective/heuristic biases in risky economic decision-making (Worthy and Maddox, 2012), and (2) overweighting low probabilities of gain or risk-seeking in loss domains (Kahneman and Tversky, 2013) (i.e., bias toward selecting from bad decks affects the rate of learning on the IGT). These neural substrates provide incremental evidence that decline in economic decision-making amongst older adults may have an organic basis.

## Data Availability Statement

The datasets generated for this study are available on request to the corresponding author.

## Ethics Statement

The studies involving human participants were reviewed and approved by Human Subjects Office, University of Iowa. The patients/participants provided their written informed consent to participate in this study.

## Author Contributions

KR conceptualized the research, analyzed the data, wrote the manuscript and assisted in data collection. KD contributed to data analyses, manuscript preparation (especially figures/tables), DT and ND contributed research and financial assistance for data collection. All subjects were recruited under the aegis of ND's aging research laboratory. All authors contributed significantly in shaping the manuscript.

## Conflict of Interest

The authors declare that the research was conducted in the absence of any commercial or financial relationships that could be construed as a potential conflict of interest.
