# Supplementary material for: The Role of Emotional vs. Cognitive Intelligence in Economic Decision-Making Amongst Older Adults
Source: Front Neurosci. 2020 May 26;14:497. doi: 10.3389/fnins.2020.00497 (PMC7274021; doi:10.3389/fnins.2020.00497)
Supplement: Supplementary file 1 [file Data_Sheet_1.PDF]

## Wilkinson-Rogers Notation for regression equations

### Table 2

**Model 1**

Iowa Gambling Task Total Score ~ Full Scale Cognitive Intelligence

+ Emotional Intelligence Index Score + Sex

**Model 2**

Iowa Gambling Task Total Score ~ Perceptual Reasoning Cognitive Intelligence

+ Verbal Comprehension Cognitive Intelligence + Experiential Emotional Intelligence

+ Strategic Emotional Intelligence + Sex

### Table 3

**Model 1**

Iowa Gambling Task Total Score ~ (1 | subject) + Emotional Intelligence Index Score + Full Scale Cognitive Intelligence + Iowa Gambling Task Block Score + Emotional Intelligence Index Score * Iowa Gambling Task Block Score + Full Scale Cognitive Intelligence * Iowa Gambling Task Block Score + Sex.

**Model 2**

Iowa Gambling Task Total Score ~ (1 | subject) + Experiential Emotional Intelligence + Perceptual Reasoning Cognitive Intelligence+ Strategic Emotional Intelligence + Verbal Comprehension Cognitive Intelligence+ Iowa Gambling Task Block Score + Experiential Emotional Intelligence * Iowa Gambling Task Block Score + Perceptual Reasoning Cognitive Intelligence * Iowa Gambling Task Block Score + Strategic Emotional Intelligence * Iowa Gambling Task Block Score + Verbal Comprehension Cognitive Intelligence* Iowa Gambling Task Block Score + Sex

### Figures 3, 4

Left Rostral Anterior Fasciculus Gyrus ~ Age

Experiential Emotional Intelligence ~ Age + Left Rostral Anterior Fasciculus Gyrus

### Figure 5

Iowa Gambling Task Total Score ~ (1 | Subject.ID.) + Sex + Right Transverse Temporal Fasciculus Gyrus * Iowa Gambling Task Block Score.
